# Kaempferol as a flavonoid induces osteoblastic differentiation via estrogen receptor signaling

**DOI:** 10.1186/1749-8546-7-10

**Published:** 2012-04-30

**Authors:** Ava Jiangyang Guo, Roy Chiyan Choi, Ken Yuzhong Zheng, Vicky Ping Chen, Tina Tingxia Dong, Zheng-Tao Wang, Günter Vollmer, David Taiwai Lau, Karl Wah-keung Tsim

**Affiliations:** 1 Division of Life Science and Center for Chinese Medicine, State Key Laboratory of Molecular Neuroscience, The Hong Kong University of Science and Technology, Clearwater Bay, New Territories, Hong Kong SAR, China; 2 Institute of Chinese Materia Medica, Shanghai University of Traditional Chinese Medicine 1200, Cailun Road, Zhangjiang Hi-Tech Park, Shanghai 201203, China; 3 Molecular Cell Physiology and Endocrinology, Technische Universität Dresden, Institut für Zoologie, Professur für Molekulare Zell-physiologie und Endokrinologie, 01062 Dresden, Germany

## Abstract

**Background:**

Flavonoids, a group of compounds mainly derived from vegetables and herbal medicines, chemically resemble estrogen and some have been used as estrogen substitutes. Kaempferol, a flavonol derived from the rhizome of *Kaempferia galanga *L., is a well-known phytoestrogen possessing osteogenic effects that is also found in a large number of plant foods.

The herb *K. galanga *is a popular traditional aromatic medicinal plant that is widely used as food spice and in medicinal industries. In the present study, both the estrogenic and osteogenic properties of kaempferol are evaluated.

**Methods:**

Kaempferol was first evaluated for its estrogenic properties, including its effects on estrogen receptors. The osteogenic properties of kaempferol were further determined its induction effects on specific osteogenic enzymes and genes as well as the mineralization process in cultured rat osteoblasts.

**Results:**

Kaempferol activated the transcriptional activity of pERE-Luc (3.98 ± 0.31 folds at 50 μM) and induced estrogen receptor α (ERα) phosphorylation in cultured rat osteoblasts, and this ER activation was correlated with induction and associated with osteoblast differentiation biomarkers, including alkaline phosphatase activity and transcription of osteoblastic genes, *e.g*., type I collagen, osteonectin, osteocalcin, Runx2 and osterix. Kaempferol also promoted the mineralization process of osteoblasts (4.02 ± 0.41 folds at 50 μM). ER mediation of the kaempferol-induced effects was confirmed by pretreatment of the osteoblasts with an ER antagonist, ICI 182,780, which fully blocked the induction effect.

**Conclusion:**

Our results showed that kaempferol stimulates osteogenic differentiation of cultured osteoblasts by acting through the estrogen receptor signaling.

## Background

Estrogen is known to play a significant role in bone metabolism in addition to its central roe in the reproductive system [1]. The osteoprotective effects of estrogen have been attributed mainly to its inhibitory action resorption of bone and stimulation of bone formation [2,3]. The drastic decrease in estrogen that accompanies menopause with the elevation of bone resoption caused by a rise in osteoclastogenesis is the most common cause of osteoporosis in women [4]. Clinically, estrogen replacement therapy has long been considered as the first-line therapy for preventing and treating osteoporosis in post-menopausal women. However, estrogen treatment is linked with an increased risk of breast and uterine cancer [4].

Flavonoids, a group of naturally occurring plant secondary metabolites that are commonly found in fruits, vegetables and Chinese herbs, have been shown to exert a protective effect against post-menopausal bone loss [5-7]. The flavonol kaempferol, which is derived from the rhizome of *Kaempferia galanga *L., has been reported to possess various biological activities. The herb *K. galanga *is a popular traditional aromatic medicinal plant used in Asian countries, including China and Japan [8]. Traditionally, kaempferol is used to treat hypertension, abdominal pains, headache, and rheumatism. Kaempferol is determined to inhibit osteoclastic bone re-sorption *in vitro *[9] and to promote differentiation and mineralization of osteoblast-like cells [10,11]. However, the precise mechanism of action of kaempferol on bone homeostasis is not clearly known.

This study aims to investigate the estrogenic and osteogenic effects of kaempferol in primary cultured osteoblasts and evaluate whether the compound has estrogenic effect, particularly osteogenesis by inducing the enzymatic activity of alkaline phosphatase (ALP), which is an indicative osteoblast differentiation marker, and transcription of osteogenesis-associated genes, including type I collagen (*COL1A1*), osteonectin and osteocalcin, and two essential transcription factors (Runx2 and osterix) in cultured cells [12]. This study will determine whether effect of kaempferol on the mineralization process of osteoblasts is mediated by estrogen receptors (ERs) and not by a Wnt/β-catenin signaling pathway.

## Materials and methods

### Chemicals

Kaempferol was purchased from Wakojunyaku (Osaka, Japan) and had a purity of over 98%. It was dissolved in dimethyl sulfoxide (DMSO) to give a stock solution of 100 mM. 17β-estradiol, ICI 182,780 and p-nitrophenyl-phosphate (pNPP) were purchased from Sigma (St. Louis, MO, USA). Recombinant human Dickkopf related protein 1 (DKK-1) and recombinant human Wnt-3a were purchased from Tocris Bioscience (Ellisville, MO, USA).

### Cell culture

Rat primary osteoblasts were cultured and prepared by a previously described method [13] with minor modifications [14]. In brief, postnatal day 1 rats were decapitated to collect calvariae. Tissues were sequential digested by 1% trypsin for 10 minutes, 0.2% collagenase for 20 minutes and another freshly prepared 0.2% collagenase for 45 minutes. The supernatant was collected after centrifugation for 5 minutes at 1500 rpm (200 × *g*). Osteoblastic cells were re-suspended and maintained in modified Eagle's medium α (MEMα), supplemented with 10% fetal bovine serum, 2 mM L-glutamine, 100 U/mL penicillin and 100 μg/mL streptomycin in a humidified CO_2 _(5%) incubator at 37°C. Before the cells were plated, they were washed with phosphate-buffered saline, and the medium was changed to MEMα (phenol red free) containing 5% charcoal dextran-treated fetal bovine serum for 2 days. The rat primary osteoblasts were then seeded and treated with different drugs at various concentrations for predetermined time periods. In pre-treatment with an ER antagonist (ICI 182,780 or DKK-1), the antagonist was first added to the cultured osteoblasts for 1 hour, and then the tested drug was applied without washing out the antagonists. Reagents for cell cultures were purchased from Invitrogen Technologies (Carlsbad, CA, USA).

### Estrogenic activity and ER phosphorylation assays

Three repeats of estrogen responsive elements (ERE: 5'-GGT CAC AGT GAC C-3') were synthesized as described previously [15,16] and then subcloned into a promoter-reporter vector pTAL-Luc (Clontech, Mountain View, CA, USA) which has a downstream firefly luciferase gene; this DNA construct was named pERE-Luc. Transient transfection of osteoblasts with the cDNA constructs was performed with Lipofectamine Plus reagent (Invitrogen, CA, USA), according to the manufacturer's instructions. Activation of luciferase gene expression driven by pERE-Luc was performed by a commercial kit (Tropix Inc., Bedford, MA, USA) [16]. The luminescent reaction was quantified in a Tropix TR717™ microplate luminometer [Applied Biosystems, Bedford, MA], and the activity was expressed as absorbance (up to 560 nm) per milligram of protein. This luciferase assay was also applied to a Wnt-responsive element, pWRE-Luc (five repeats of GAT CAA A) that was used here to test Wnt-induced signaling [12]. The phosphorylation of ERα (at serine 118) was determined by Western blot. Cultures of primary osteoblasts were serum starved for 3 hours prior to the addition of kaempferol. After treatment, the cultures were collected immediately in lysis buffer containing 125 mM Tris-HCl (pH 6.8), 2% sodium dodecyl sulfate (SDS), 10% glycerol and 200 mM 2-mercaptoethanol, and the proteins were subjected to SDS-PAGE analysis. After transfer, the membrane was incubated with anti-phospho-ERα-S118 antibody (1:2000; Upstate, Lake Placid, NY, USA) and anti-total ERα antibody (1:1000; Upstate) at 4°C for 12 hours for protein detection. The immuno-complexes were visualized and quantified by the enhanced chemiluminescence method (GE Healthcare) as described previously [12,15].

### ALP and mineralization assays

Treated osteoblasts were placed in lysis buffer containing 0.2% Triton X-100, 1 mM dithiothreitol and 100 mM potassium phosphate buffer (pH 7.8). ALP activity was measured by mixing the cell extract with 5 mM pNPP (Sigma, St. Louis, MO, USA) in a buffer (pH 10.4) containing 0.1 M glycine, 1 mM MgCl_2 _and 1 mM ZnCl_2 _at 37°C, and measuring the absorbance at 405 nm. In the mineralization analysis, cultured osteoblasts were cultured for 21 days. Treatment with kaempferol (10 μM) or 17β-estradiol (100 nM) in the presence of β-glycerophosphate (20 ng/mL) was performed at 3-day intervals. After 21 days of culture, the cells were rinsed with deionized water twice and fixed in 70% ice-cold ethanol for 1 hour at 4°C. The mineralization assay was performed by staining the cells with 4% Alizarin Red S (Sigma, St. Louis, MO, USA) for 15 minutes at room temperature and washing them five times with deionized water. The stained cells were then dehydrated with 70% ethanol followed by absolute ethanol. Cells were observed with phase contrast microscope at a magnification of 20×, and orange-red staining indicated the position and intensity of the calcium deposits. Alizarin red was quantified as previously described [12].

### Real-time quantitative PCR

Total RNA from cultured osteoblasts was isolated by RNAzol^®^RT reagent (Molecular Research Center, Cincinnati, OH, USA), and 5 μg of RNA was reverse-transcribed by Moloney murine leukemia virus reverse transcriptase (Invitrogen, CA, USA), according to the manufacturer's instructions. Real-time PCR of COL1A1 (234 bp), osteonectin (182 bp), osteocalcin (281 bp), Runx2 (252 bp), osterix (159 bp) and 18 S rRNA (320 bp) transcripts was performed on equal amounts of reverse-transcribed products, using KAPA™ SYBR^® ^FAST qPCR kit according to the manufacturer's instructions (Kapa Biosystems, Cape Town, South Africa). The primers were designed according to the database from genebank (NM_053304 for COL1A1; NM_012656 for osteonectin; NM_013414 for osteocalcin; NM_001146038.1 for Runx2; NM_130458.3 for osterix and NR_003286 for 18 S rRNA. The SYBR green signal was detected by a Mx3000p™ multiplex quantitative PCR machine (Stratagene, La Jolla, CA, USA). The relative levels of transcript expression were quantified by using the ΔΔCt method [17]. The calculation was done by using the Ct value of 18 S rRNA to normalize the Ct value of the target gene in each sample to obtain the ΔCt value, which then was used for comparison among different samples. The PCR products were analyzed by gel electrophoresis, and the specificity of amplification was confirmed by the melting curve.

### Protein assay

Protein concentrations were measured routinely by Bradford's method with a kit from Bio-Rad Laboratories (Hercules, CA, USA).

### Statistical analysis

Independent *t*-test was carried out with SPSS software (version 13.0, SPSS, Chicago, IL, USA). *P *values were corrected by the Bonferroni method for multiple comparison. The level of statistical significance was *P *< 0.05.

## Results and discussion

**Figure 1:**
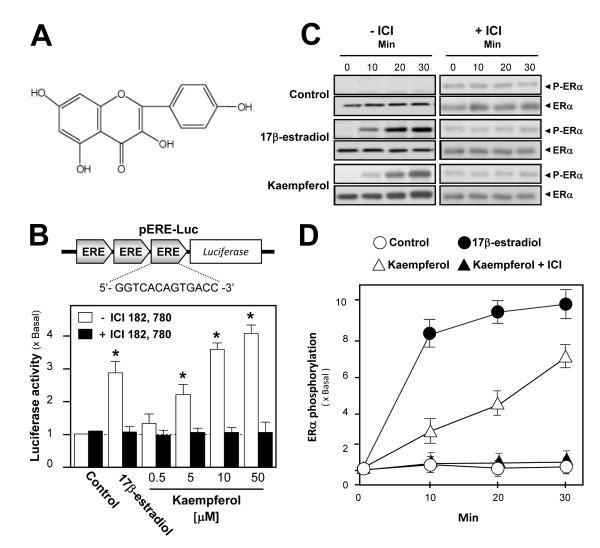
**Estrogenic activities of kaempferol in cultured osteoblasts**. **A: **The chemical structure of kaempferol. **B: **Three repeats of ERE were tagged with a luciferase reporter gene to form pERE-Luc (upper panel). The DNA construct pERE-Luc was stably transfected into cultured osteoblasts, which were then treated with kaempferol at various concentrations or 17β-estradiol (1 nM) with or without an hour of pre-treatment with ICI 182,780 (100 nM). After 48 hours of treatment, luciferase activity was determined. As compared to DMSO control, statistically significant results include the effects of 17β-estradiol (*P *= 0.041), 10 μM (*P *= 0.0425) and 50 μM (*P *= 0.038). **C: **Cultured osteoblasts were treated with kaempferol (10 μM) and 17β-estradiol (1 nM) with or without pre-treatment with ICI 182,780 (100 nM) for 1 hour. Lysates were subjected to Western blot analysis to determine the phosphorylation of ERα at serine 118 and total ERα. **D: **The signals were quantified from the blots in (C) by calibrated densitometry. Values in all panels are expressed as the fold increase from basal reading (control culture; 0.02% DMSO) and are in mean ± SD, n = 5, each with triplicate samples.

ERs are the members of the superfamily of ligand-regulated nuclear transcription factors. ERα and ERβ, have been identified in cultured rat osteoblasts [12] and estrogen was shown to stimulate the differentiation of osteoblasts (Additional file 1). Thirty-six flavonoids, mainly derived from vegetables and Chinese herbs, were screened for their ability to actively stimulate osteoblast differentiation [12], and kaempferol (Figure 1A) was one of the positive hits. The estrogenic activity of the kaempferol was determined by its induction effect on pERE-Luc-transfected cultured osteoblasts (Figure 1B, upper panel). 17β-estradiol was used as the positive control and induced pERE-Luc activity about 3-fold upon treatment (*P *= 0.041). In osteoblasts expressing pERE-Luc, kaempferol induced luciferase activity in a dose-dependent manner: the luciferase activity increased to 3.98 ± 0.31 folds (at 50 μM, *P *= 0.038) after treatment as shown in Figure 1B. These activities showed the authenticity of pERE-Luc construct. Kaempferol treatment, even at rather high concentrations, did not affect cell viability; therefore, toxic side effects within the dose range investigated can be excluded (Additional file 2). In addition, pre-treatment with ICI 182,780 fully blocked kaempferol-induced pERE-Luc activity, indicating that kaempferol acts via ER activation. Kaempferol was able to trigger ERα (~66 kDa) phosphorylation at the S118 position in a time-dependent manner in cultured osteoblasts, generating a 7-folds increase at 30 minutes of treatment (Figures 1C and 1D), which could serve as further evidence of its estrogenic property. 17β-estradiol served as a positive control with a nearly 10-folds increase in ERα phosphorylation at 30 minutes, while ICI 182,780 completely blocked kaempferol-induced ERα phosphorylation. In all cases, the total amount of ERα remained unchanged.

**Figure 2:**
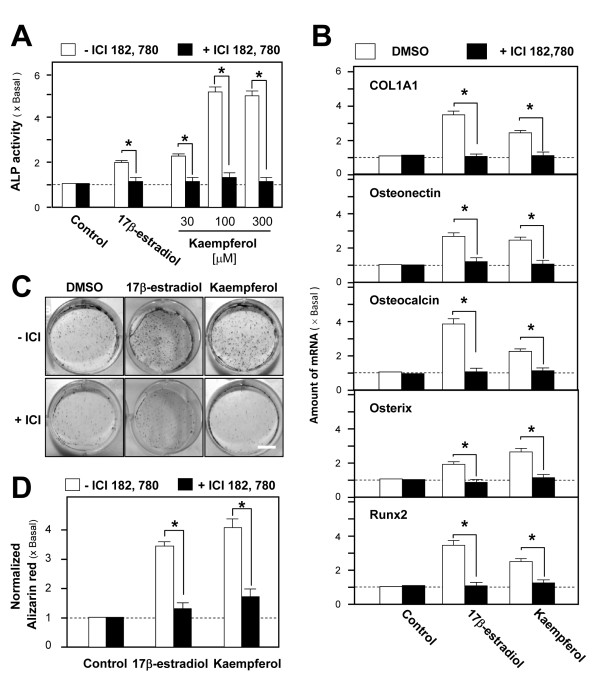
**Kaempferol-induced osteogenic differentiation is mediated by ER signaling cultured osteoblasts**. **A: **Application of 17β-estradiol (100 nM) or kaempferol (30 - 300 μM) in cultured osteoblasts for 3 days increased ALP activity in a dose-dependent manner. The stimulatory effect was abolished upon pre-treatment with ICI 182,780 (100 nM) for 1 hour. The ALP activities detected after the pre-treatment of ICI 182, 780 were compared with the ALP activities detected without the pre-treatment. The statistically significant results include the blocking effects of 17β-estradiol (*P *= 0.0412), kaempferol at 30 μM (*P *= 0.0485), 100 μM (*P *= 0.0081) and 300 μM (*P *= 0.0086). **B: **Cultured osteoblats were treated with 17β-estradiol (100 nM) or kaempferol (10 μM) for 2 days, with or without pre-treatment with ICI 182,780 (100 nM) for 1 hour. Total RNAs were extracted from the cultures to perform quantitative PCR for osteogenesis-associated genes, including type I collagen (*COL1A1*), osteonectin, osteocalcin, osterix and Runx2 mRNAs. The mRNA amounts of osteogenesis-associated genes detected after the pre-treatment of ICI 182, 780 were compared with the mRNA amounts detected without the pre-treatment. The statistically significant results include the blocking effects of 17β-estradiol (*P *= 0.0012 for COL1A1; *P *= 0.0070 for osteonectin; *P *= 0.0033 for osteocalcin; *P *= 0.0441 for osterix and *P *= 0.0023 for Runx2) and kaempferol (*P *= 0.0065 for COL1A1; *P *= 0.0063 for osteonectin; *P *= 0.0072 for osteocalcin; *P *= 0.0068 for osterix and *P *= 0.0064 for Runx2). **C: **Cultured osteoblasts underwent mineralization upon the addition of 17β-estradiol (100 nM) or kaempferol (10 μM) in the presence of β-glycerophosphate (5 mM). After 21 days of treatment, nodules were found, as shown by Alizarin Red staining. The mineralization process was hindered by pre-treatment with ICI 182,780 (100 nM). **D: **From the cultures of (C), Alizarin Red staining was quantified using a solution of 20% methanol and 10% acetic acid in water, and the reading was done on a spectrophotometer at 450 nm. The normalized alizarin red amounts detected after the pre-treatment of ICI 182, 780 were compared with the amount detected without the pre-treatment. The statistically significant results include the blocking effects of 17β-estradiol (*P *= 0.0093) and kaempferol (*P *= 0.0085). Values in all panels are expressed as the fold increase from the basal reading (control culture; 0.02% DMSO); mean ± SD, n = 5, each with triplicate samples.

The effects of kaempferol on osteoblastic differentiation were determined in cultured osteoblasts. Application of kaempferol in the cultures induced ALP activity in a dose-dependent manner (Figure 2A): 2.08 ± 0.29 folds induction was reached with about 30 μM kaempferol. In comparison with the ALP activity induced by 17β-estradiol (1.98 ± 0.19 folds increase), the effect of kaempferol was more robust. Osteoblastic ALP activities induced by both 17β-estradiol and kaempferol were fully blocked by ICI 182,780 (Figure 2A, P = 0.0412 and *P *= 0.0485, respectively). Transcription of genes for several bone differentiation markers, (COL1A1, osteonectin, osteocalcin, Runx2 and osterix, was up-regulated with 17β-estradiol and kaempferol treatment in cultured osteoblasts. In all cases, the transcripts encoding these markers were markedly induced 3 to 4-folds by 100 nM 17β-estradiol and 2 to 3-folds by 10 μM kaempferol. The inductions were completely blocked by pre-treatment with ICI 182,780 (Figure 2B). The statistically significant results include the blocking effects of 17β-estradiol (*P *= 0.0012 for COL1A1; *P *= 0.0070 for osteonectin; *P *= 0.0033 for osteocalcin; *P *= 0.0441 for osterix and *P *= 0.0023 for Runx2) and kaempferol (*P *= 0.0065 for COL1A1; *P *= 0.0063 for osteonectin; *P *= 0.0072 for osteocalcin; *P *= 0.0068 for osterix and *P *= 0.0064 for Runx2). In addition, both 17β-estradiol and kaempferol induced osteoblastic mineralization (3.27 ± 0.34 folds and 4.02 ± 0.41 folds, respectively), which was fully blocked by the treatments with ICI 182,780 (Figures 2C and 2D). The kaempferol results were similar to those of the 17β-estradiol treatment, suggesting that the osteogenic property of kaempferol was entirely due to its estrogenic properties. The statistically significant results include the blocking effects of 17β-estradiol (*P *= 0.0093) and kaempferol (*P *= 0.0085).

**Figure 3:**
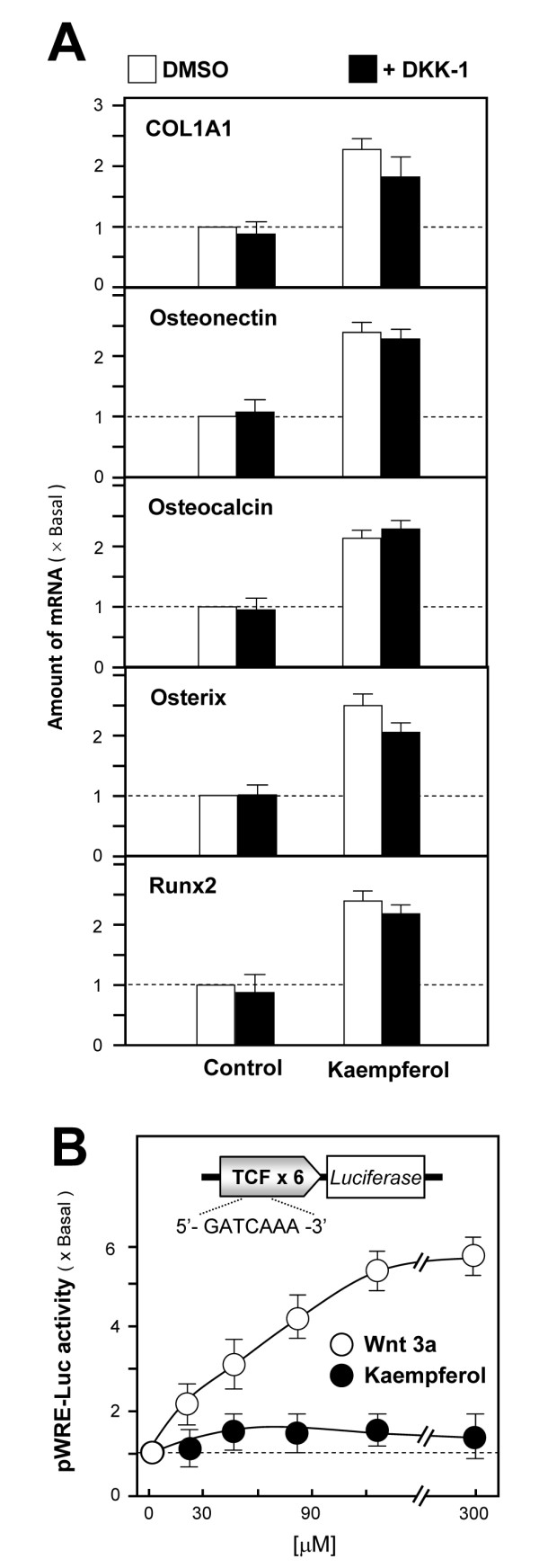
**Kaempferol-stimulated osteogenic effect is not mediated by activation of the Wnt/β catenin pathway**. **A: **Cultured osteoblasts were treated with kaempferol (10 μM) for 2 days, with or without DKK-1 pre-treatment (0.2 mg/mL) for 1 hour. Total mRNA was extracted from the cultures to perform quantitative PCR for osteogenesis-associated genes, including type I collagen (*COL1A1*), osteonectin, osteocalcin, Runx2 and osterix. **B: **A reporter construct corresponding to pWRE-Luc (upper panel) was used as described previously. pWRE-Luc was transfected into cultured osteoblasts for 2 days before addition of Wnt3a (200 ng/mL; the ligand of Wnt/β-catenin pathway) or kaempferol (10 μM). Forty-eight hours later, luciferase activity was assayed. Values are expressed as the fold increase from the basal reading (control culture; 0.02% DMSO); mean ± SD, n = 5, each with triplicate samples.

Furthermore, pre-treatment with DKK-1, a Wnt receptor inhibitor, could not block kaempferol-induced effects (Figure 3A). The specific inhibition by ICI 182,780 (Figure 2B) but not by DKK-1 confirmed that kaempferol-induced osteogenic effects were mediated by the activation of ERα via a classical ER signaling pathway. In addition, kaempferol did not show any activation of Wnt/β-catenin signaling, as tested by a luciferase reporter pWRE-Luc (Figure 3B). All evidence collectively indicates that kaempferol-induced osteoblast differentiation is mediated by ER signaling.

As an estrogen alternative without the associated adverse effects of the hormone, flavonoids, a large group of naturally occurring compounds with estrogen-like activities and a valuable potential source for new dietary health interventions for post-menopausal women, have been intensively investigated for their ability in preventing post-menopausal bone loss [18]. Flavonoids have long been recognized to possess a wide range of biological activities [19], and the mechanisms for these activities are being actively explored. Kaempferol is known to be the most abundant phytoestrogen in Western diets as compared with soybean isoflavones [18]. This compound is widely found in many food plants [11,20].

Previously, we have shown that the flavone baicalin, which is derived from the roots of *Scutellaria baicalensis*, possesses stimulatory effects on osteoblast differentiation [12]. This baicalin-induced bone effect was not mediated by its estrogenic property. Rather, baicalin promoted osteogenesis via regulation of the Wnt/β-catenin signaling cascade. In the present study, kaempferol was shown to enhance osteoblastic differentiation and mineralization via ER signaling by inducing ERα phosphorylation and transcriptional activity of ERE. Unlike baicalin, kaempferol did not activate the Wnt/β-catenin pathway, and the kaempferol-induced osteogenic effects could not be abolished upon treatment with the Wnt receptor inhibitor DKK-1. Taken together, these results suggested that the osteogenic effects mediated by flavonoids could be dependent or independent of their estrogenic properties. This idea is supported by our previous screening of different flavonoids, encompassing the major sub-classes, which showed that the estrogenic activities of selective flavonoids are not correlated with osteogenic activities [12]. Moreover, our results also implied that different flavonoids could promote bone differentiation via different signaling mechanisms and these mechanisms of action and signaling cascades should be further studied. Further, combining flavonoids that regulate bone differentiation via different mechanisms could have synergistic or additive effects, which could be further verified. The potential of kaempferol as a bone-promoting flavonoid should be investigated for developing potential drugs or food supplements for the prevention of the bone loss associated with menopause.

## Conclusion

Our results showed that kaempferol stimulates osteogenic differentiation of cultured osteoblasts by acting through the estrogen receptor signaling.

## Abbreviations

ALP: Alkaline phosphatase; COL1A1: Type I collagen; DMSO: Dimethyl sulfoxide; DKK-1: Dickkopf related protein 1; ER: Estrogen receptor; ERE: Estrogen responsive element; PNPP: P-nitrophenyl-phosphate; Runx2: Runt-related transcription factor 2.

## Competing interests

The authors declare that they have no competing interests.

## Authors' contributions

KT, RC, TD, ZW, and GV designed the study and wrote the manuscript. AG conducted the experiments and drafted the manuscript. KZ and VC assisted in performing the experiments. All authors read and approved the final version of the manuscript.

## Supplementary Material

Additional file 1 **Figure 1**. Treatment of kaempferol did not affect the cell viability of cultured osteoblasts. Cultured osteoblasts were challenge with 7 β-estradiol (10 nM), or different doses of kaempferol for 2 day, and the viability was determined by MTT assay. Values are expressed as the % of the control reading (control cultured treated with 0.02% DMSO), and are in mean ±, where n = 4, each with triplicate samples. Click here for file

Additional file 2 **Figure 2**. Estrogen induces osteogenic effect in cultured rat osteoblasts. The osteogenic expressions of estrogen receptors was determined in cultured rat osteoblasts. Click here for file
