# Supplementary material for: Kaempferol as a flavonoid induces osteoblastic differentiation via estrogen receptor signaling
Source: Chin Med. 2012 Apr 30;7:10. doi: 10.1186/1749-8546-7-10 (PMC3350445; doi:10.1186/1749-8546-7-10)
Supplement: Additional file 1 — Figure 1. Treatment of kaempferol did not affect the cell viability of cultured osteoblasts. Cultured osteoblasts were challenge with 7 β-estradiol (10 nM), or different doses of kaempferol for 2 day, and the viability was determined by MTT assay. Values are expressed as the % of the control reading (control cultured treated with 0.02% DMSO), and are in mean ±, where n = 4, each with triplicate samples. [file 1749-8546-7-10-S1.PDF]

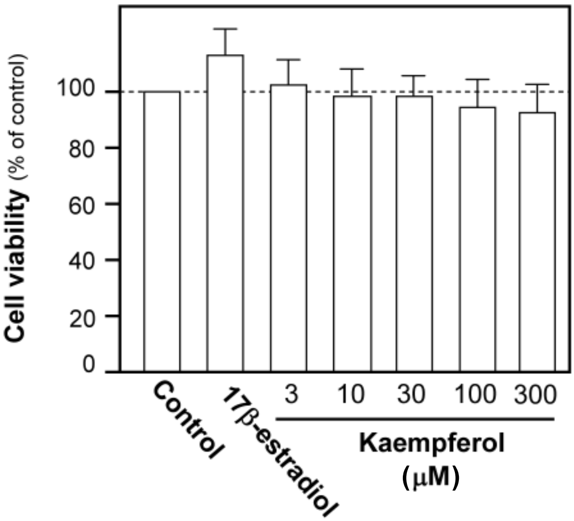

Supplementary Figure 1: Treatment of kaempferol did not affect the cell viability of cultured osteoblasts. Cultured osteoblasts were challenged with 17β-estradiol (10 nM), or different doses of kaempferol for 2 days, and the cell viability was determined by MTT assay. Values are expressed as the % of the control reading (control culture treated with 0.02% DMSO), and are in mean ± SD, where *n*=4, each with triplicate samples.
