# Supplementary material for: Kaempferol as a flavonoid induces osteoblastic differentiation via estrogen receptor signaling
Source: Chin Med. 2012 Apr 30;7:10. doi: 10.1186/1749-8546-7-10 (PMC3350445; doi:10.1186/1749-8546-7-10)
Supplement: Additional file 2 — Figure 2. Estrogen induces osteogenic effect in cultured rat osteoblasts. The osteogenic expressions of estrogen receptors was determined in cultured rat osteoblasts. [file 1749-8546-7-10-S2.PDF]

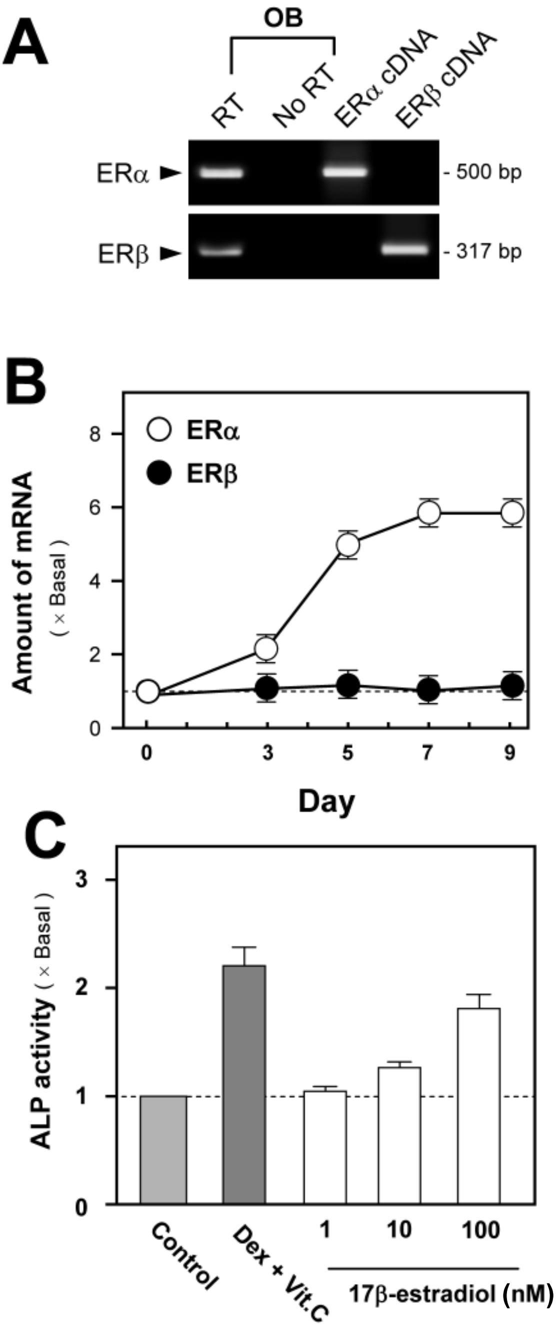

Supplementary Figure 2: Estrogen induces osteogenic effect in cultured rat osteoblasts. The osteogenic expressions of estrogen receptors was determined in cultured rat osteoblasts. **A:** Total RNAs were extracted from cultured osteoblasts to perform PCR for ERα (500 bp) and ERβ (317 bp) using specific primers. PCR products were resolved on a 1% SYBR safe stained-agarose gel and visualized under the UV light. ERα and ERβ cDNAs served as positive controls. Representative images are shown, *n*=3. **B:** The expression profiles of ERα and ERβ during the differentiation of cultured osteoblasts were determined by quantitative real-time PCR upon the application of dexamethasone and vitamin C. **C:** 17β-estradiol induces ALP activity in cultured osteoblasts. The cultures were treated with 17β-estradiol at different doses as indicated, for 3 days, and the ALP assay was performed. Dexamethasone and vitamin C served as the positive control. Values are expressed as the fold of increase to basal reading (control culture treated with 0.02% DMSO), and are in mean $\pm$ SD, where *n*=3, each with triplicate samples.
